# Molecular characterization of viruses associated with encephalitis in São Paulo, Brazil

**DOI:** 10.1371/journal.pone.0209993

**Published:** 2019-01-14

**Authors:** Jerenice E. Ferreira, Suzete C. Ferreira, Cesar Almeida-Neto, Anna S. Nishiya, Cecilia S. Alencar, Gisele R. Gouveia, Helio Caiaffa-Filho, Helio Gomes, Raimunda Telma de Macedo Santos, Steven S. Witkin, Alfredo Mendrone-Junior, Ester C. Sabino

**Affiliations:** 1 LIM / 46—Laboratory of Medical Parasitology, Institute of Tropical Medicine—IMT, University of São Paulo Medical School USP, São Paulo, Brazil; 2 Pathology Center, Adolf Lutz Institute–IAL, São Paulo, Brazil; 3 Fundação Pró-Sangue Hemocentro de São Paulo–FPS/HSP, São Paulo, Brazil; 4 Disciplina de Ciências Médicas da Faculdade de Medicina da Universidade de São Paulo, São Paulo, Brazil; 5 Central Laboratory Division—Hospital das Clínicas São Paulo–DLC-HCSP; 6 Clinical Laboratory and LIM 03–Department of Pathology, Clinical Hospital, University of São Paulo Medical School, São Paulo, Brazil; 7 Faculty of Medicine, University of São Paulo–FMUSP, São Paulo, Brazil; 8 Department of Obstetrics and Gynecology, Weill Cornell Medicine, New York, NY, United States of America; Defense Threat Reduction Agency, UNITED STATES

## Abstract

The objective of this study was to characterize the prevalence of viral encephalitis due to arbovirus infection of the Togaviridae and Flaviviridae families in São Paulo, Brazil. A total of 500 cerebrospinal fluid (CSF) samples collected between August 2012 and January 2013, from patients with symptoms of acute encephalitis were analyzed. Findings suggestive of viral encephalitis—elevations in cell concentration, glucose and total protein—were observed in 234 (46.8%) samples, designated as Group 1. The remaining 266 samples comprised Group 2. All samples were tested for Flaviviruses (dengue virus 1, 2, 3 and 4, yellow fever virus and West Nile virus), Alphavirus (NS5 region) and enterovirus by RT- PCR and for herpesviruses and enteroviruses using CLART^-^Entherpex. A presumptive viral etiological agent was detected in 26 samples (5.2%), 18 (8.0%) in Group 1 and 8 (3.0%) in Group 2. In Group 1 human herpesviruses were detected in 9 cases, enteroviruses in 7 cases, dengue viruses (DENV) in 2 CSFs and St. Louis encephalitis virus (SLEV) in one case. In Group 2 there were 3 CSFs positive for human herpesviruses, 2 for enteroviruses, 2 for DENV and 1 for SLEV. Detection of arboviruses, even though present in a minority of infected patients, identifies these viruses as a probable etiological agent of encephalitis. This is of special concern in regions where this class of viruses is endemic and has been linked to other recent epidemics.

## Introduction

Encephalitis is associated with high morbidity and mortality, as well as with cognitive, behavioral and even symptomatic epilepsies [1]. Approximately 60% of patients suffer from severe memory disorder, which presents as clinical dementia [1]. In addition to the personal, family and social cost, if these patients survive, they rarely become productive again [1,2]. The incidence of encephalitis ranges from 1.5 to 8/100,000 inhabitants, being higher in children under one year of age [3,4].

Viruses known to cause encephalitis worldwide are herpesviruses, arboviruses and enteroviruses [1]. Arboviruses are the leading cause of encephalitis worldwide [2]. In developed countries, herpes simplex virus type 1 (HSV-1) is the leading cause of sporadic encephalitis in adults, while Varicella zoster virus (VZV) is responsible for up to 22% of pediatric cases [1].

Overall, Japanese encephalitis virus (JEV) is the most prevalent arbovirus associated with neurological disease based on total number of annual cases [1].

In the West, the West Nile virus (WNV) is the most frequent agent of arbovirus encephalitis and can be found in parts of Europe, Russia, Africa, the Middle East, India, Indonesia and North America [1]. There are few studies evaluating the prevalence of encephalitis-related viruses in Brazil [4]. Similar to other countries, the most frequent cause of viral encephalitis in Brazil appears to be HSV- 1, followed by arboviruses [4]. Bastos et al., identified the presence of herpesviruses, enteroviruses and arboviruses in CSF samples from 165 patients suspected of central nervous system viral infection by gene amplification [5].

Among arboviruses circulating in Brazil, members of the Togaviridae, Flaviviridae, Peribunyaviridae, Reoviridae and Rhabdoviridae families cause the highest number of infections and diseases in humans [4]. Bussuquara virus (AROAV), Cacicaporé virus (CPCV), dengue virus (DENV) serotypes 1–4, Rocio virus (ROCV), Iguape virus (IGUV), Ilhéus virus (ILHV), yellow fever virus (YFV) and Saint Louis encephalitis virus (SLEV) have also been reported at low frequency in Brazil [6,7]. Chikungunya virus (CHIKV) was first reported in Central America in late 2013, and by the end of 2014 was present in most countries in South America [8–13]. Zika virus (ZIKV) was first reported in Brazil in 2014, but clinical cases were only recognized in mid-2015 in the states of Bahia and Rio Grande do Norte, located in the Northeast of Brazil [8–13].

In Brazil there exists an elevated risk for the emergence and/or spread of new arboviruses to humans [14]. This country has large urban centers with a high population density that are infested by mosquitoes of the genus *Culex* and *Aedes* that transmit arboviruses to man [14]. The prevalence and dispersion of arboviruses has rapidly increased globally in recent years coinciding with improvements in global transportation, adaptation of its arthropod vectors to urbanization, failure to contain increases in the mosquito population and deforestation [14]. These arthropods apparently rapidly adapt to enhanced exposure to humans and domestic animals [14].

The primary objective of this study was to assess the prevalence of sporadic, non-seasonal viral encephalitis associated with arbovirus infection of the Togaviridae and Flaviviridae families in patients in São Paulo, Brazil. A second objective was to ascertain the prevalence of herpesviruses and enteroviruses in these patients and to identify the distribution of cases by gender, age, locality and characteristics of the cerebrospinal fluid (CSF).

## Materials and methods

### Clinical study design and sample collection

A cross-sectional study was performed from August 2012 to January 2013. A total of 500 cerebrospinal fluid samples were collected at the Hospital das Clínicas of the University of São Paulo Medical School (HCFMUSP) from individuals (both genders and any age group) with clinical suspicion of acute encephalitis (at least two of the following symptoms: fever, altered level of consciousness, headache, focal neurological deficits and seizure). HCFMUSP is the largest public hospital complex in Latin America, and is a center of tertiary care. It comprises eight institutes and two auxiliary hospitals with more than two thousand and one hundred beds [15, 16]. The distribution of encephalitis cases were from the metropolitan region, coastal and inland regions of São Paulo state as well as from other states in Brazil (Acre, Amazonas, Bahia, Ceará, Maranhão, Pará and Paraná). Suspicious patients were referred to the Special Analysis / Liquor Department of the Central Laboratory of HCFMUSP, where the aspiration of CSF was performed from the lumbar region of the spinal cord. Collected material was sent for biochemical, microbiological and cytological analysis. From each sample collected, 1 mL was set aside for molecular investigation of viral agents. Research on human subjects was conducted after Hospital das Clínicas—University of São Paulo’s Institutional Review Board (CAPPesq) approval, online registration #8874/12. All patients provided written informed consent prior to CSF collection. In the case of patients under 18 years old, written consent was obtained of their parents or legal representatives.

Patients were classified into two groups according to CSF cellularity, glucose and total protein concentration. Group 1 included patients with pleocytosis (cellularity >4 cells/mm3) comprised predominantly of lymphocytes, glucose >50 mg/dL and protein >10 mg/dL. When one or more of these parameters were absent, patients were designated as Group 2 [17].

Physical characteristics of CSF were performed by visual observation of color and appearance before and after centrifugation. CSF biochemistry was performed by an automated method using the Modular Analytics System (Roche—Switzerland). The cytological analysis of CSF was performed by manually using a Fucks-Rosenthal camera for global counts of red blood cells, leukocytes and tissue cells. A differential leukocyte count was performed by CytospinTM (Cytospin TM 4 Cytocentrifuge—Thermo Fisher Scientific—Massachusetts, USA). The pellet fraction was affixed to slides and staining was performed using the Hemogram Slide Ink equipment (Vyttra Diagnósticos—São Paulo—Brazil) and a Colorgram kit (Wright-Giemsa-Leishman).

### Molecular detection

#### Extraction of nucleic acids

Viral RNA in CSF was extracted with the Qiagen UltraSens kit (QIAmpUltraSens—QiagenGmbH, Hilden, Germany) using an initial volume of 250 μl according to the manufacturer's protocol. Two μl of canine adenovirus (DNA- CAV Vanguard HTLP 5 / CV-L 86-4230-65) at a 1/100 dilution was added as an exogenous internal control.

#### D-RT-PCR

Amplification of viral RNA was performed using the Duplex Reverse Transcription-PCR (D-RT-PCR) for detection and Identification of Alphaviruses and Flaviviruses, as described previously [18].

#### Real Time PCR

The quantitative real-time PCR reactions were performed according to the protocols Callahan et al., for DENV serotypes 1, 3 and 4 [19]; Johnson et al., for DENV serotype 2 [20]; Mantel et al., for yellow fever virus- YFV [21]; and Briese et al., for WNV in the NS5 region [22].

Real–time PCR for enteroviruses was performed according to a methodology described by Volle et al. [23]. The CLART^-^Entherpex (BioMérieux—Ivry-sur-Seine, France) kit was used to identify eight human herpesviruses: HSV-1, herpes simplex virus type 2 (HSV-2), VZV, cytomegalovirus (CMV), Epstein- Barr virus (EBV), human herpesvirus 6 (HHV-6), human herpesvirus 7 (HHV-7) and human herpesvirus 8 (HHV-8) and the three major enteroviruses, poliovirus (PV), echovirus (E) and coxsackievirus (CV). The methodology is based on the detection of the amplification by RT-PCR of a fragment located between 106–328 pb, which is detected by a low density micro-matrix platform (Microarray).

#### Sequencing

Samples of CSF in which virus was detected by D-RT-PCR were submitted to sequencing analysis according to the method of Sanger. The sequencing reaction was performed using the kit ABI PRISM BigDye Terminator Cycle Sequencing Ready Reaction (Applied Biosystems, Life Technologies, Carlsbad, California, United States) according to the manufacturer's instructions. Sequencing was completed using the Genetic Analyzer ABI 3100 (Applied Biosystems, Life Technologies, Carlsbad, California, U.S).

The sequences were analyzed using the program Sequencher 3.0 Sequencing Software (Ann Arbor, Michigan, United States) and compared with the genomic database GenBank (National Center for Biotechnology Information—NCBI- Bethesda, Maryland, U.S).

### Statistical analysis

The statistical analyses, chi-square test, were performed using Epi Info version 7.2 (Centers for Disease Control and Prevention (CDC), Atlanta, Georgia, United States) and Excel from Office 2010 (Redmond, Washington, United States).

## Results and discussion

Among 500 patients with a clinical suspicion of encephalitis, 234 (46.8%) of their CSF samples were positive for an increased concentrations of cells, protein and glucose. These patients, whose CSF findings were suggestive of viral encephalitis, were designated as Group 1. The remaining 266 subjects comprised Group 2. Identification of a possible viral etiologic agent of encephalitis was accomplished in only 18 (8.0%) CSF samples from patients in Group 1 and 8 (3.0%) from those in Group 2. Table 1 summarizes the demographic characteristics of patients according to rates of virus detection. Gender was equally divided between male and female and the largest groups of patients were those over 50 years old. There were no differences in either category between those positive or negative for viruses or between Groups 1 and 2.

**Table 1:** Gender and age of patients with suspected viral encephalitis.

Data	Group 1				Group 2			
(Variables)	Positive cases	Negative cases	Total	*p value*	Positive cases	Negative cases	Total	*p value*
	N = 18 (8.0%)	N = 216 (92.0%)	N = 234 (100%)		N = 8 (3.0%)	N = 258 (97.0%)	N = 266 (100%)	
**Gender**								
Male	11 (5.0%)*	92 (39.0%)	103 (44.0%)	0.128	04 (1.5%)	133 (50.0%)	137 (51.5%)	0.931
Female	07 (3.0%)	124 (53.0%)	131 (56.0%)		04 (1.5%)	125 (47.0%)	129 (48.5%)	
**Age**								
0–14	06 (2.7%)	14 (5.9%)	20 (8.6%)	0.000704	01 (0.4%)	19 (7.1%)	20 (7.5%)	0.926
15–29	01 (0.5%)	33 (14.0%)	34 (14.5%)		02 (0.75%)	53 (19.9%)	55 (20.7%)	
30–49	03 (1.3%)	79 (33.7%)	82 (35.0%)		02 (0.75%)	81 (30.5%)	83 (31.2%)	
> 50	08 (3.5%)	90 (38.4%)	98 (41.9%)		03 (1.1%)	105 (39.5%)	108 (40.6%)	

*One patient had concomitant positive results for EV (enterovirus) and HHV-6. Chi-square test.

Table 2 summarizes the viruses detected in both groups. In Group 1 members of the herpesvirus family predominated (9 cases) followed by enteroviruses (7 cases). Three arbovirus infections, two of DENV and one case of SLEV were also identified. In Group 2 an equal number of herpesvirus and arbovirus (2 DENV, 1 SLEV) infections were detected (3 cases each), followed by enteroviruses (2 cases).

**Table 2:** Molecular etiology of positive cases of viral encephalitis.

Data	Arboviruses N (%)		Herpesvirus N (%)					Enterovirus	Unknown
(Variables)	DENV	SLEV	HSV-1	EBV	CMV	HHV-6	VZV	N (%)	etiology
Group 1	2 (0.8%)	1 (0.4%)	1 (0.4%)	4 (1.7%)	1 (0.4%)	3 (1.3%)*	0 (0.0%)	7 (3.0%)*	216 (92.0%)
(N = 234)									
Group 2	2 (0.8%)	1 (0.4%)	0 (0.0%)	0 (0.0%)	1 (0.4%)	1 (0.4%)	1 (0.4%)	2 (0.8%)	258 (97.0%)
(N = 266)									

*One patient had concomitant infection with EV (enterovirus) and HHV-6 (Real Time PCR and CLART^-^Entherpex). DENV: dengue virus (D-RT-PCR and Real Time PCR); SLEV: Saint Louis encephalitis virus (D-RT-PCR); HSV-1: Herpes Simplex virus type 1 (CLART^-^Entherpex); EBV: Epstein-Barr virus (CLART^-^Entherpex); CMV: Cytomegalovirus (CLART^-^Entherpex); HHV-6: Human herpesvirus 6 (CLART^-^Entherpex); VZV: Varicella zoster virus (CLART^-^Entherpex) [20–25].

The two positive SLEV CSFs were both from women, 32- and 40- years-old, living in metropolitan São Paulo. One presented with facial palsy while the other had an uneventful clinical course without any adverse sequelae. Both SLEV samples underwent nucleotide sequencing to confirm their identity. The fragment obtained for the initial analysis was 232 bp. The sample from the Group 1 patient was 98% similar, and for the Group 2 patient 95% identical to SLEV isolated from the BsAsARG-10 NS5 gene (Sequence ID: gb JQ003910.1).

Table 3 shows the cellularity, biochemistry and physical characters of CSF among patients in Groups 1 and 2 who tested positive for virus. These variables could not distinguish between the different viruses identified in either group.

**Table 3:** CSF data from patients in Groups 1 and 2 who tested positive for virus.

	Enterovirus		Herpesvirus		SLEV		DENV	
	Group 1	Group 2	Group 1	Group 2	Group 1	Group 2	Group 1	Group 2
Cellularity								
Lymphomononuclear								
> 10%	7 (3.0%)	1 (0.4%)	9 (3.8%)	1 (0.4%)	1 (0.4)	–	2 (0.8%)	1 (0.4%)
<10%	–	1 (0.4%)	–	2 (0.8%)	–	1 (0.4%)	–	1 (0.4%)
Erythrocytes								
1–99 cells/mm^3^	7 (3.0%)	1 (0.4%)	9 (3.8%)	2 (0.8%)	1 (0.4)	1 (0.4%)	2 (0.8%)	1 (0.4%)
> 100 cells/mm^3^	–	1 (0.4%)	–	1 (0.4%)	–	–	–	1 (0.4%)
Biochemistry								
Glucose								
> 50 mg/dL	7 (3.0%)	–	9 (3.8%)	2	1 (0.4)	1 (0.4%)	2 (0.8%)	–
< 50 mg/dL	–	2 (0.8%)	–	1 (0.4%)	–	–	–	2 (0.8%)
Protein								
> 10 mg/dL	7 (3.0%)	2 (0.8%)	9 (3.8%)	2	1 (0.4)	1 (0.4%)	2 (0.8%)	2 (0.8%)
< 10 mg/dL	–	–	–	1 (0.4%)	–	–	–	–
Physical Characters								
Clear and Colorless	4 (1.7%)	1 (0.4%)	7 (3.0%)	3 (1.1%)	1 (0.4%)	1 (0.4%)	2 (0.8%)	2 (0.8%)
Clear and Xantochromic	1 (0.4%)	–	2 (0.8%)	–	–	–	–	–
Slightly Hemorrhagic	–	–	–	–	–	–	–	–
Slightly turbid or cloudy	2 (0.8%)	1 (0.4%)	–	–	–	–	–	–
Turbid or cloudy	–	–	–	–	–	–	–	–

SLEV: Saint Louis encephalitis virus.

The prevalence of arbovirus-related encephalitis in subjects in our study was 2.4%, including 4 cases of DENV and 2 of SLEV. This occurrence is lower than what has been reported in other investigations [5]. Conversely, the identification of herpesviruses and enteroviruses as the most frequent CSF isolates from our patients is consistent with prior studies [5,24,25]. However, rates of detection and the specific herpesviruses that were present differed between our study and other investigations. Recent studies in New Orleans reported a 20.4% prevalence of HSV and a 5.1% prevalence of VZV in encephalitis patients [25]. In the present study HHV-6 and EBV were the most prevalent herpesviruses viruses detected. Divergent population characteristics between São Paulo and New Orleans as well as differences in socio-economic levels, seasonal weather variations and alterations in mosquito-related variables could account for these variations [25,26].

In Brazil, three strains of SLEV have been isolated from the blood of patients who did not display neurological symptoms. Two patients from Pará, in the North of the country, presented with fever and jaundice, and one from São Paulo had clinical symptoms consistent with dengue fever [27–29]. Non-human SLEV infections have also been found in southeastern Brazil, where the virus was isolated from wild birds, rodents and sentinel mice during a surveillance program of arbovirus activity carried out in the state of São Paulo from 1967 to 1969 [27–29]. In a study performed in cities in the Brazilian Amazon (Bragança and Salvaterra / Pará, Macapá / Amapá and Rio Branco / Acre) and in Maracaju, Mato Grosso do Sul in the midwest of the country, immunity to SLEV was identified in horses. No SLEV-associated disease was apparent in these animals [29,30]. However, in a study carried out in Minas Gerais, Brazil, SLEV was found in the brain of a horse with neurological symptoms and its identity was confirmed by molecular and serological analysis [31]. SLEV is also considered as a possible etiological agent in human cases of dengue-like illness in São Paulo [27,32]. Recently, SLEV was detected by molecular techniques in four serum samples from patients with clinical suspicion of dengue and in two patients with suspected viral meningoencephalitis, all from the Municipality of São José do Rio Preto, State of São Paulo [33]. In the study by Heinen et al., SLEV was detected in three patients co-infected with DENV serotype 4 in the cities of Cuiabá and Várzea Grande, one of whom had triple co-infection with DENV subtype1 [4,34–37]. The small number of identified SLEV infections in people in Brazil is likely due to the occurrence of asymptomatic infections as well as difficulty in the differential diagnosis of individual flaviviruses [5,27,32]. However, these studies are suggestive of a wide distribution of SLEV in Brazil [29].

The arrival of the WNV has been expected in Brazil since its appearance in the United States in 2000 [38–43]. Acknowledging the limits of detection, our data suggest that this virus is not yet a cause of encephalitis in São Paulo.

Epidemics of DENV and ZIKV have been recent occurrences in Brazil and they are considered to be the most important flaviviruses circulating in this country [4,34]. Consequently, laboratory diagnosis during epidemic periods is directed at detection of these viruses. This hinders the detection of other circulating flaviviruses, including SLEV, which may silently becoming widely dispersed in the Americas [4,33–36]. Therefore, our results indicate the need for a wider spectrum of active surveillance of arboviruses during outbreaks of dengue [4,34].

In the present study no presumptive viral agent was identified in 90% of suspected cases of encephalitis. Worldwide, >70% of cases of presumptive encephalitis cases are not associated with any known infectious agent. Limitations and sensitivity of diagnostic methods, timing of sample collection and storage conditions, viral load and evolution of the disease in infected individuals are factors that may decrease ability to detect etiologic agents [2,5, 44–46]. In addition, a non-infectious etiology, due to sterile inflammation, autoimmunity or environmental exposures, remains an additional possibility.

This outbreak of 500 presumed encephalitis cases within a six month period was not registered at the São Paulo State Health Department most likely due to underreporting. This absence of official notification increases the likelihood that public health officials will be under-prepared to initiate measures to reduce exposure to causative agents, including arboviruses [27].

## Conclusions

Although we found only a low prevalence of arboviruses in this encephalitis population, there remains a need to employ molecular tests to identify this class of viruses in patients with encephalitis. This is critical in our opinion due to epidemics caused by related flaviviruses in Brazil. Continuous monitoring of arbovirus as a cause of encephalitis is essential for the adequate treatment of these patients as well as for emphasizing a need for the implementation of sanitary measures to reduce the population of their mosquito vectors. The detection of different viruses in the central nervous system of patients with meningitis or encephalitis highlights the importance of maintaining a diagnostic laboratory capable of identifying a variety of viruses with high sensitivity [5]. Detection of the specific causative virus will improve clinical decision making and the choice of the most appropriate antiviral therapy [5].

 
